# Intrapancreatic fat deposition is unrelated to liver steatosis in metabolic dysfunction-associated steatotic liver disease

**DOI:** 10.1016/j.jhepr.2023.100998

**Published:** 2024-01-01

**Authors:** Anne Linde Mak, Nienke Wassenaar, Anne-Marieke van Dijk, Marian Troelstra, Veera Houttu, Koen van Son, Stan Driessen, Diona Zwirs, Sandra van den Berg-Faay, Elizabeth Shumbayawonda, Jurgen Runge, Michail Doukas, Joanne Verheij, Ulrich Beuers, Max Nieuwdorp, Djuna L. Cahen, Aart Nederveen, Oliver Gurney-Champion, Adriaan Holleboom

**Affiliations:** 1 Department of Vascular Medicine, Amsterdam University Medical Centers, Amsterdam, The Netherlands; 2 Amsterdam Gastroenterology Endocrinology Metabolism (AGEM) Institute, Amsterdam UMC, University of Amsterdam, Amsterdam, The Netherlands; 3 Department of Radiology and Nuclear Medicine, Amsterdam University Medical Centers, Amsterdam, The Netherlands; 4 Cancer Center Amsterdam, Imaging and Biomarkers, Amsterdam, The Netherlands; 5 Department of Gastroenterology and Hepatology, Radboudumc, Nijmegen, The Netherlands; 6 Perspectum Ltd., Oxford, United Kingdom; 7 Department of Radiology, Netherlands Cancer Institute, Amsterdam, The Netherlands; 8 Department of Pathology, Erasmus Medical Center, Rotterdam, The Netherlands; 9 Department of Pathology, Amsterdam University Medical Centers, Amsterdam, The Netherlands; 10 Department of Gastroenterology and Hepatology, Amsterdam University Medical Centers, Amsterdam, The Netherlands; 11 Department of Gastroenterology and Hepatology, Erasmus University Medical Center, Rotterdam, The Netherlands

**Keywords:** fatty pancreas disease, pancreatic steatosis, MASLD, MASH, multiparametric MRI

## Abstract

**Background & Aims:**

Individuals with obesity may develop intrapancreatic fat deposition (IPFD) and fatty pancreas disease (FPD). Whether this causes inflammation and fibrosis and leads to pancreatic dysfunction is less established than for liver damage in metabolic dysfunction-associated steatotic liver disease (MASLD). Moreover, the interrelations of FPD and MASLD are poorly understood. Therefore, we aimed to assess IPFD and fibro-inflammation in relation to pancreatic function and liver disease severity in individuals with MASLD.

**Methods:**

Seventy-six participants from the Amsterdam MASLD-MASH cohort (ANCHOR) study underwent liver biopsy and multiparametric MRI of the liver and pancreas, consisting of proton-density fat fraction sequences, T1 mapping and intravoxel incoherent motion diffusion-weighted imaging (IVIM-DWI).

**Results:**

The prevalence of FPD was 37.3%. There was a clear correlation between pancreatic T1 relaxation time, which indicates fibro-inflammation, and parameters of glycemic dysregulation, namely HbA1c (*R* = 0.59; *p <*0.001), fasting glucose (*R* = 0.51; *p <*0.001) and the presence of type 2 diabetes (mean 802.0 ms *vs*. 733.6 ms; *p <*0.05). In contrast, there was no relation between IPFD and hepatic fat content (*R* = 0.03; *p* = 0.80). Pancreatic IVIM diffusion (IVIM-D) was lower in advanced liver fibrosis (*p <*0.05) and pancreatic perfusion (IVIM-*f*), reflecting vessel density, inversely correlated to histological MASLD activity (*p <*0.05).

**Conclusions:**

Consistent relations exist between pancreatic fibro-inflammation on MRI and endocrine function in individuals with MASLD. However, despite shared dysmetabolic drivers, our study suggests IPFD is a separate pathophysiological process from MASLD.

**Impact and implications:**

Metabolic dysfunction-associated steatotic liver disease (MASLD) is the most common chronic liver disease worldwide and 68% of people with type 2 diabetes have MASLD. However, fat infiltration and inflammation in the pancreas are understudied in individuals with MASLD. In this cross-sectional MRI study, we found no relationship between fat accumulation in the pancreas and liver in a cohort of patients with MASLD. However, our results show that inflammatory and fibrotic processes in the pancreas may be interrelated to features of type 2 diabetes and to the severity of liver disease in patients with MASLD. Overall, the results suggest that pancreatic endocrine dysfunction in individuals with MASLD may be more related to glucotoxicity than to lipotoxicity.

**Clinical trial number:**

NTR7191 (Dutch Trial Register).

## Highlights

• The prevalence of fatty pancreas disease in individuals with MASLD is 37.3%.
• There is no cross-sectional correlation between intrapancreatic and intrahepatic fat deposition in individuals with MASLD.
• Pancreatic fibro-inflammation, as measured on MRI, correlates with parameters of glycemic dysregulation.

## Graphical abstract

**Figure:**
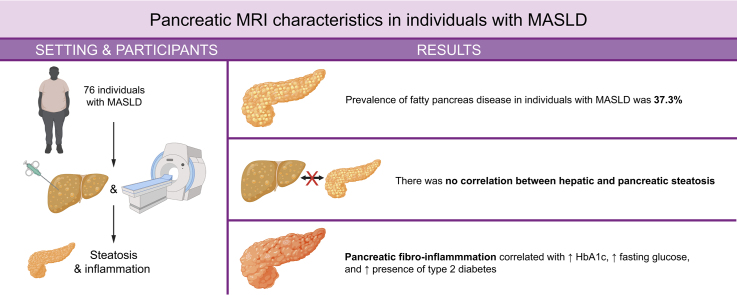

## Introduction

Fatty pancreas disease (FPD) is characterized by excessive intrapancreatic fat deposition (IPFD). This can occur within pancreatic cells, both in endocrine and acinar cells (intra-lobular fat), as well as through extracellular infiltration of adipocytes (inter-lobular fat).^1^^,^^2^ FPD has also been referred to as pancreatic steatosis, fatty pancreas, or NAFPD (non-alcoholic fatty pancreas disease).^1^^,^^3^ A meta-analysis of pancreatic fat content on MRI found that IPFD of up to 6.2% can be considered normal, setting this as the threshold for FPD in future studies.^4^ It is still unknown whether FPD causes inflammation and fibrosis and ultimately leads to pancreatic dysfunction or even malignancy, similar to disease progression in metabolic dysfunction-associated steatotic liver disease (MASLD) and metabolic dysfunction-associated steatohepatitis (MASH).^5^

MASLD and FPD have overlapping risk factors and dysmetabolic drivers.^3^ MASLD, previously known as non-alcoholic fatty liver disease (NAFLD), affects an estimated 30% of the global population.^6^ It is considered the hepatic component of the metabolic syndrome. In MASLD development, insulin resistance plays a central role. In patients with type 2 diabetes, the prevalence of MASLD increases up to 68%,^7^ and individuals with MASLD are at a twofold increased risk of incident type 2 diabetes within 5 years.^8^

The relationship between FPD and glucose homeostasis is less clear.^9^^,^^10^ Studies have shown higher IPFD in individuals with type 2 diabetes compared to healthy individuals,^11^ and conversely, an increased prevalence of type 2 diabetes in patients with FPD.^4^ However, an inverse relationship between IPFD and beta-cell function was only seen in patients with impaired glucose tolerance and not in healthy individuals.^12^ This finding led to the term glucolipotoxicity, describing the hypothesis that damage due to IPFD only occurs in a high glucose environment.^9^ A large meta-analysis of studies reporting on the prevalence of FPD in various metabolic disorders showed that neither BMI nor waist circumference was associated with FPD.^4^

To date, conflicting data have been published on the relationship between MASLD and FPD.^10^ While several studies have reported a positive association,[13], [14], [15] there are also cohorts in which no association was found.^16^^,^^17^ A study of 43 patients with biopsy-proven MASLD illustrated that IPFD measured by MRI correlated with hepatic steatosis grade on histology. However, when hepatic fibrosis was present, pancreatic fat content was lower.^18^ Della Corte *et al.* evaluated the presence of FPD in children with MASLD using ultrasound and found that those with FPD had more advanced stages of liver disease, *i.e.* higher fibrosis, ballooning and NAFLD activity scores (NAS) on histopathological evaluation.^19^ Similarly, results from a retrospective cohort study of 104 adults with biopsy-proven MASLD show that IPFD on ultrasound was predictive of advanced hepatic fibrosis.^20^ Studying the relationship between FPD and MASLD severity using multiparametric MRI in a larger cohort will aid in clarifying the interrelations between FPD and MASLD in the setting of obesity and insulin resistance.

Quantitative MRI offers an extensive set of tools to assess both the liver and the pancreas non-invasively.^21^^,^^22^ We recently showed good performance of multiparametric MRI of the liver compared to liver biopsy in patients with MASLD, and importantly, in distinguishing MASH from simple steatosis.^21^ MRI proton-density fat fraction (PDFF) accurately reflects hepatic and pancreatic steatosis.[21], [22], [23] T1 mapping enables evaluation of the MRI relaxation properties of a tissue and is a measure of fibro-inflammatory disease activity.^24^ T1 relaxation time has been shown to increase significantly with the severity of chronic pancreatitis.^24^^,^^25^ Finally, intravoxel incoherent motion diffusion-weighted imaging (IVIM-DWI) enables the measurement of diffusion and micro-vascular properties of tissues. IVIM diffusion (IVIM-D) values increase when water molecules can diffuse more freely through tissue, for example in edema due to increased vascular permeability. IVIM-D values decrease when the diffusion of water molecules is restricted by cellular elements, blood or dense fibrosis.^26^ IVIM perfusion fraction (IVIM-*f*) has been shown to correlate with vessel density in pancreatic ductal adenocarcinoma specimens.^27^

The aim of our study was thus to perform a multiparametric MRI assessment of the pancreas by PDFF, T1 mapping, and IVIM-DWI, to investigate the relation between the pancreas and liver in individuals with MASLD. We hypothesized that patients with MASLD encounter disruptions in pancreatic function due to FPD and pancreatic fibro-inflammation and that these pancreatic processes interrelate with liver disease severity. Here, we evaluate the presence of FPD in individuals with histologically characterized MASLD and correlate MRI parameters of the pancreas to liver imaging and histology scores. Moreover, we relate our pancreatic imaging findings to pancreatic endocrine and exocrine function.

## Patients and methods

### Cohort

Patients were derived from the previously described ANCHOR (Amsterdam MASLD-MASH cohort) study.^21^ This prospective observational cohort study includes individuals with hepatic steatosis detected by abdominal ultrasound or FibroScan, elevated transaminase levels and a BMI >25 kg/m^2^. Individuals with excessive alcohol use (women >14 units/week, men >21 units/week), with detectable causes of hepatic steatosis other than MASLD, or with known bleeding disorders or anticoagulant use were excluded. Included individuals underwent multiparametric MRI of the liver and pancreas, as well as an ultrasound-guided liver biopsy. The ANCHOR study was approved by the Medical Research Ethics Committee of the Amsterdam UMC and is registered in the Dutch Trial Register under number NTR7191. All participants provided written informed consent and the study was conducted in compliance with the principles of the Declaration of Helsinki.

### Liver biopsies

Percutaneous ultrasound-guided liver biopsies were performed within 2 weeks after the MRI scan under local anesthesia with a 17- or 18-gauge biopsy needle by a hepatologist or interventional radiologist at the Amsterdam UMC, following local standard procedure. Biopsies were stained with H&E and picrosirius red and scored in tandem by two expert liver pathologists (M.D. and J.V.) according to the SAF (steatosis, activity, and fibrosis) score.^28^ Using the SAF score, steatosis grade, inflammatory activity grade (*i.e*. lobular inflammation + hepatocyte ballooning) and fibrosis stage are scored individually.

### MRI sequences

A clinical 3.0T MRI unit (Ingenia; Philips, Best, the Netherlands) with a 16-channel phased-array anterior coil and a 10-channel phase-arrayed posterior coil was used for multiparametric MRI. Participants fasted overnight before scanning, and all data were acquired in a single scanning session. Magnitude-based PDFF was used to quantify hepatic steatosis and IPFD. PDFF was determined using a multi-echo gradient echo sequence with six echo times. The LiverMultiScan® protocol (Perspectum Ltd, Oxford, UK), described elsewhere,^29^ was used for T1 mapping. Four transverse slices positioned at the porta hepatis were captured using a shMOLLI (shortened modified look-locker inversion) to quantify liver and pancreas T1. An IVIM-DWI sequence was used as a proxy for inflammation and fibrosis and consisted of a free-breathing multi-slice diffusion-weighted single-shot echo-planar imaging sequence with 18 unique b-values. Details of the MRI acquisition parameters are given in Table 1.

**Table 1:** MRI acquisition parameters.

	PDFF	LiverMultiScan	IVIM-DWI
Field of view (mm^3^)	448 × 320 × 180	440 × 330 × 100	450 × 295 × 188
Reconstruction voxel size (mm^2^)	2 × 2	1.15 × 1.15	1.8 × 1.8
Slice thickness (mm)	5	8	6
Slice gap (mm)	0	7 (for liver)	1
Slices	36	5 for liver, 1 for pancreas	27
Parallel imaging SENSE factor	1.5	2	1.3
Repetition time (ms)	150	2.42	7000
Echo time (ms)	1.15, 2.33, 3.51, 4.69, 5.86, 7.04	1.05	46
Flip angle (°)	10	35	90
Acquisition duration	18 s	60 s for liver, 12 s for pancreas	8:10 min
b-values (sec/mm^2^) and [number of averages]	—	—	0 [9], 1 [3], 2 [3], 5 [3], 10 [3], 20 [3], 30 [3], 40 [3], 50 [3], 75 [3], 100 [3], 150 [3], 200, 300, 400 [3], 500 [3], 600 [3], 700 [3]
Respiratory compensation	1 breath-hold	5 breath-holds for liver, 1 breath-hold for pancreas	Free breathing
Fat saturation	—	—	Gradient reversal during slice selection + SPAIR

cT1, corrected T1; IVIM-DWI, intravoxel incoherent motion diffusion-weighted imaging; PDFF, proton-density fat fraction.

### Image analysis

To analyze the PDFF of the liver, three regions of interest (ROIs) were placed in three different slices, maximizing their size while avoiding large vessels, bile ducts and liver edges. The mean signal intensity per TE was then determined. Similarly, for PDFF analysis of the pancreas, three ROIs of 100 mm^2^ were placed in the head, body and tail regions, avoiding organ edges. Subsequently, a multi-echo and multifrequency water and fat signal model enabled correction for T2∗ effects and was used to calculate the PDFF of both organs.^30^ The mean PDFF values of all three ROIs of each organ were averaged to establish the fat percentage of the liver and pancreas.

T1 map reconstruction was performed using LiverMultiScan® software.^29^ T1 was scanner referenced for field strength to obtain srT1. Subsequently, the liver T1 maps were corrected for iron (cT1) and the mean cT1 value of the liver (excluding large vessels) was calculated. During the study, we extended the field of view to include the pancreas, obtaining a subset of individuals in which T1 of the pancreas could also be evaluated. For this subset analysis, three circular ROIs were placed in the head, body and tail of the pancreas (one in each region) on single transverse T1 maps.^31^

For IVIM-DWI analyses, the diffusion (IVIM-D) and perfusion fraction (IVIM-*f*) were calculated using an unsupervised physics-informed deep neural network in Python using Pytorch, which has been described previously.^32^^,^^33^ The whole pancreas was delineated on the reconstructed DWI images (average over all b-values). The mean IVIM-*f* and IVIM-D were reported.

### Collection of blood and stool samples

Blood samples were collected on the morning of the MRI after an overnight fast. All blood analyses except C-peptide and pro-insulin were performed by the clinical chemistry library of the Amsterdam UMC upon blood withdrawal. Separate blood tubes were processed for storage at -80 °C to be analyzed at a later date. C-peptide was measured in stored heparinized plasma using an immunoluminometric assay on an automated immunoanalyzer (Atellica IM, Siemens). Pro-insulin was measured in stored EDTA plasma using the Human Total Proinsulin ELISA kit by Millipore. Moreover, patients collected morning stool samples which were stored at -80 °C until the measurement of fecal elastase levels by ELISA (BIOSERV Diagnostics). Patients were categorized based on fecal elastase results into either normal exocrine function (≥200 μg/g) or exocrine insufficiency (<200 μg/g).

### Statistical analyses

Statistical analyses were conducted using R version 4.2.1. Shapiro-Wilk tests were performed to check the normality of the data. For continuous variables, either Pearson or Spearman correlation tests were used to assess linear correlations, depending on the normality of the data distribution. For categorical data, *t* tests or Mann-Whitney *U* tests were used to compare two groups, and ANOVA or Kruskall-Wallis tests were used to compare multiple groups, depending on the normality of the data distribution. A *p* value of <0.05 was considered statistically significant.

We first checked whether outcomes of pancreatic imaging (*i.e*. PDFF, T1 and IVIM) were influenced by age, BMI or type 2 diabetes status. If significant differences were seen in pancreatic imaging between individuals with and without type 2 diabetes, we further related the imaging outcome to HbA1c and fasting glucose, insulin, pro-insulin to insulin ratio (PIR) and C-peptide levels, as well as the C-peptide to insulin molar ratio.^34^ Next, we related pancreatic imaging outcomes to liver imaging (*i.e*. PDFF and cT1) as well as to liver histology scoring for steatosis, inflammatory activity and fibrosis (F ≤2 *vs.* F ≥3).

## Results

### Participants

Seventy-six participants were included in the analyses and MRI of the liver and pancreas was performed in all. The pancreas was not fully captured on PDFF imaging in one participant. In a subgroup of 38, T1 mapping of the pancreas was also available. Baseline characteristics are shown in Table 2. Notably, participants were relatively young (average age 47) and hyperinsulinemic (median fasting insulin 121.5 pmol/L). The cohort consisted of 31 female and 45 male participants. Besides significantly higher alkaline phosphatase levels in female participants, there were no major differences in characteristics between the sexes (Table S1). All patients had hepatic steatosis, and at least one cardiometabolic criterium for MASLD (Table S2).

**Table 2:** Baseline characteristics.

	All participants (n = 76)	Pancreatic T1 subgroup (n = 38)
Age	47.4 (13.6)	48.6 (13.2)
Sex (male/female)	45/31 (59.2%/40.8%)	17/21 (44.7%/55.3%)
BMI (kg/m^2^)	32.78 [29.47, 36.07]	31.27 (4.23)
Type 2 diabetes (%)	31 (40.8)	16 (42.1)
HbA1c (mmol/mol)	40.5 [36.0, 53.0]	40.0 [36.0, 47.3]
Fasting glucose (mmol/L)	6.1 [5.5, 7.8]	6.0 [5.4, 7.6]
Fasting insulin (pmol/L)	121.5 [78.3, 175.8]	114.5 [73.0, 160.5]
AST (U/L)	42 [35, 57]	41 [35, 57]
ALT (U/L)	62 [48, 91]	62 [47, 103]
GGT (U/L)	62 [38, 92]	67 [37, 93]
ALP (U/L)	86 (31)	89 (33)
**Histology scoring of MASLD**		
Steatosis		
S0	3 (3.9%)	2 (5.3%)
S1	23 (30.3%)	10 (26.3%)
S2	30 (39.5)%	17 (44.7%)
S3	20 (26.3%)	9 (23.7%)
Inflammatory activity		
A0	4 (5.3%)	2 (5.3%)
A1	21 (27.6%)	10 (26.3%)
A2	36 (47.4%)	17 (44.7%)
A3	14 (18.4%)	9 (23.7%)
A4	1 (1.3%)	0 (0.0%)
Fibrosis		
F0	3 (3.9%)	0 (0.0%)
F1	9 (11.8%)	7 (18.4%)
F2	39 (51.3%)	18 (47.4%)
F3	19 (25.0%)	10 (26.3%)
F4	6 (7.9%)	3 (7.9%)

ALP, alkaline phosphatase; ALT, alanine aminotransferase; AST, aspartate aminotransferase; GGT, gamma-glutamyltransferase; MASLD, metabolic dysfunction-associated liver disease.

Hepatic steatosis, lobular inflammation and fibrosis are graded according to the SAF scoring system.^28^ Data are presented as mean (SD), median [IQR], or count (percentage).

### Descriptive statistics of pancreatic imaging

#### Pancreatic fat fraction

Pancreatic fat percentage, as measured by PDFF, ranged from 0.3 to 19.4% (median 4.6, IQR 3.3–8.4). When using the suggested cut-off of 6.2%,^4^ 28 out of 75 individuals (37.3%) had pancreatic steatosis. The pancreatic fat fraction did not increase with age or BMI (Fig. S1A), nor did it differ between participants with and without type 2 diabetes (Fig. S1B). When comparing pancreatic regions, the fat fractions descended slightly from head to body to tail, but this difference was not significant (Fig. S1C).

#### Pancreatic T1

Pancreatic T1 relaxation time ranged from 603 to 1,133 ms (median 758; IQR 707–815) and was not related to age or BMI (Fig. S2).

**Fig. 1:**
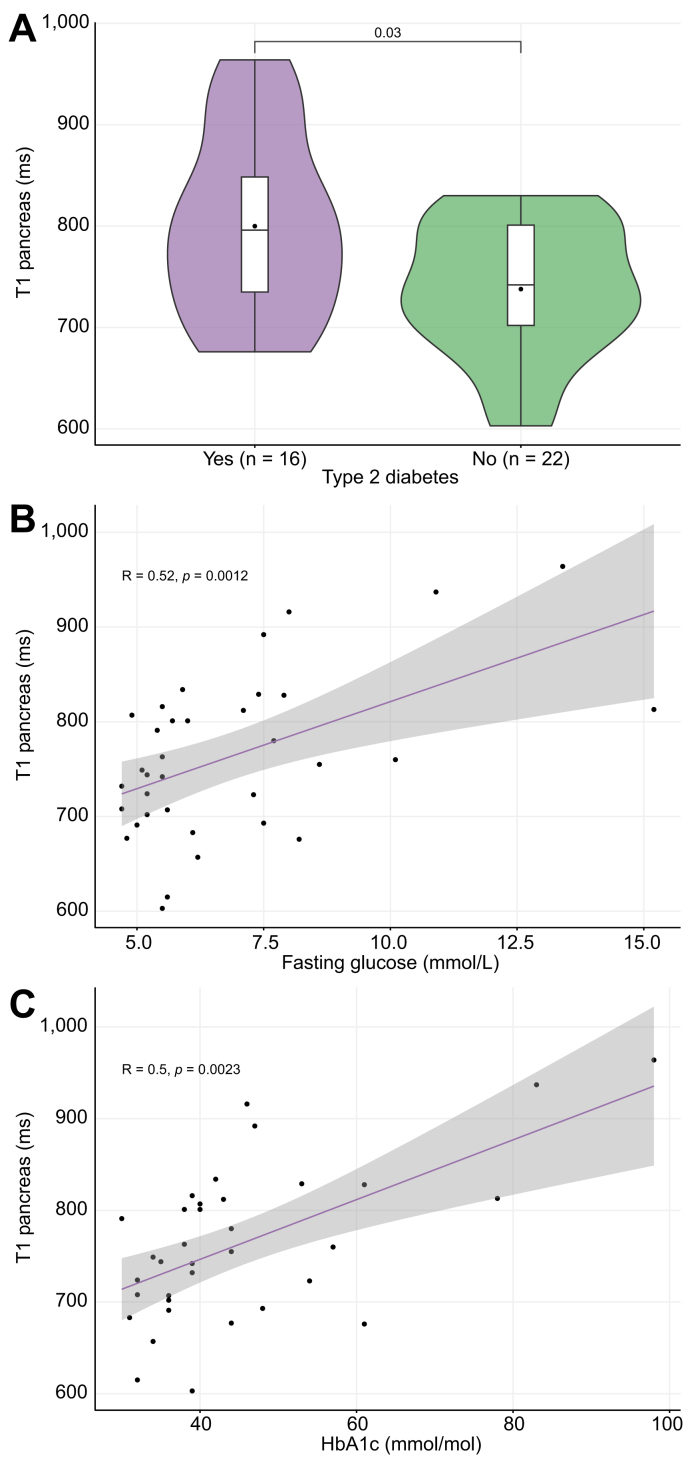
The T1 relaxation time of the pancreas is higher in individuals with impaired glucose control. (A) Type 2 diabetes. Level of significance: *p* = 0.03 (t-test). (B) Fasting glucose levels. Level of significance: *p* = 0.0012 (Spearman’s rho). (C) HbA1c. Level of significance: *p* = 0.0023 (Spearman’s rho).

Pancreatic T1 did not correlate with pancreatic fat fraction (*R* = 0.16, *p* = 0.34). However, it was significantly higher in participants with type 2 diabetes than in those without (mean 799.9 ms *vs*. 737.9 ms, respectively; *p <*0.05; Fig. 1A). Moreover, pancreatic T1 correlated linearly to fasting glucose (*R* = 0.52, *p <*0.01; Fig. 1B) and HbA1c (*R* = 0.5, *p <*0.01; Fig. 1C). When excluding outliers, defined as points at 1.5x IQR below quartile 1 and above quartile 3, results remained significant (fasting glucose *p <*0.05; HbA1c *p <*0.05). There was no relation between pancreatic T1 and fasting insulin levels (*R* = 0.12, *p* = 0.5) or PIR (*R* = −0.02, *p* = 0.92). A trend towards an inverse correlation was found between pancreatic T1 and C-peptide levels (*R* = −0.33, *p* = 0.05). There was no correlation between pancreatic T1 and the C-peptide to insulin molar ratio, as a measure of insulin clearance (*R* = −0.065, *p* = 0.72).

#### Pancreatic IVIM-DWI

The range in IVIM-D values was 1.01–1.68x e^−3^ mm^2^/s (mean 1.31x e^−3^ mm^2^/s, SD 0.14; normal mean ranges between 1.02–1.94x e^−3^ mm^2^/s^26^). The range in IVIM-*f* values was 8.6–28.8% (median 13.3%, IQR 11.7–15.1%; normal mean 23.7% SD 5^25^). Neither IVIM-D nor IVIM-*f* was significantly influenced by age, BMI or type 2 diabetes status.

### Pancreatic imaging in relation to liver imaging

#### Pancreatic fat fraction

**Fig. 2:**
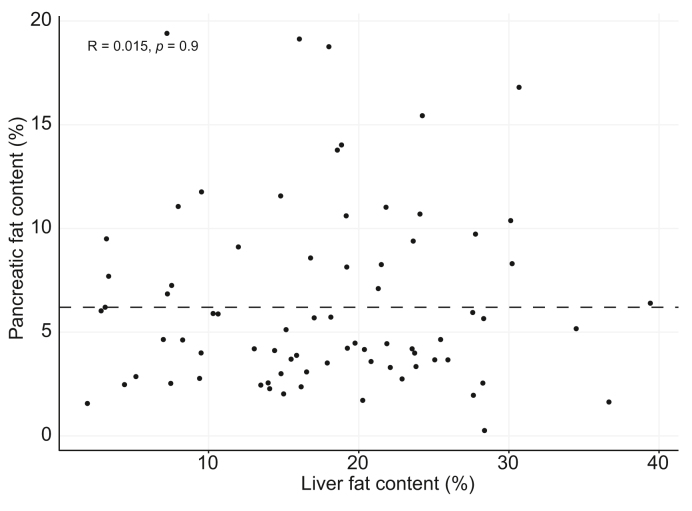
Fat content of the pancreas is not related to the fat content of the liver. Both pancreatic and liver fat content are displayed as assessed by PDFF. Level of significance: *p* = 0.9 (Spearman’s rho). Dashed line represents the FPD cut-off of 6.2%. FPD, fatty pancreas disease; PDFF, proton-density fat fraction.

Pancreatic PDFF was not correlated to liver PDFF (Spearman’s Rho, *R* = 0.02, *p* = 0.90) (Fig. 2).

#### Pancreatic T1

There was a trend towards decreasing pancreatic T1 relaxation time with an increase in liver PDFF values, though this was not statistically significant (*R* = −0.28, *p* = 0.09). Hepatic cT1 values, as a surrogate marker for fibro-inflammation in the liver, did not correlate with pancreatic T1 (*R* = −0.08, *p* = 0.65).

#### Pancreatic IVIM-DWI

Pancreatic IVIM-D slightly decreased with increasing liver PDFF (*R* = −0.24, *p* = 0.04). There was no correlation between pancreatic IVIM-D and cT1 of the liver. Pancreatic IVIM-*f* values did not correlate with either liver PDFF or liver cT1.

### Pancreatic imaging in relation to liver histology

#### Pancreatic fat fraction

**Fig. 3:**
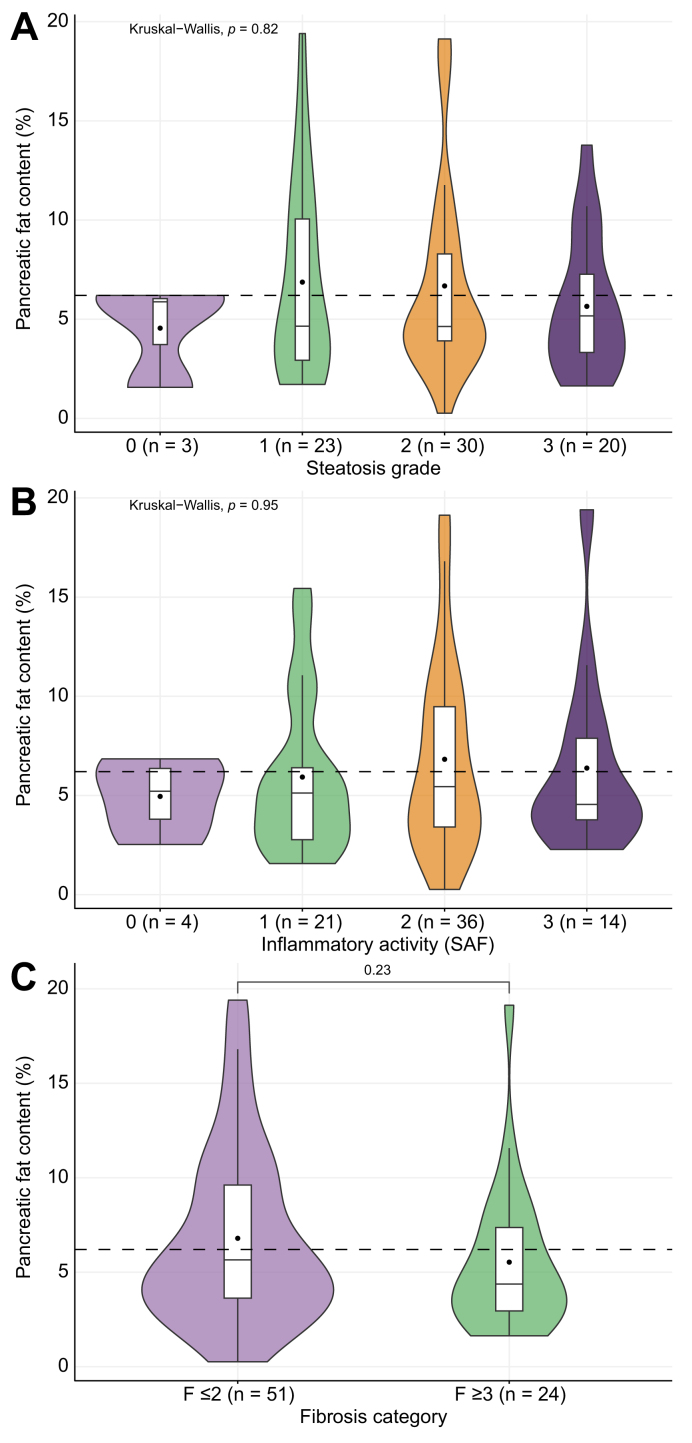
Relations between pancreatic fat content and liver histology scores. (A) Pancreatic fat content is unrelated to liver steatosis grade. Level of significance: *p* = 0.82 (Kruskall-Wallis test). (B) Pancreatic fat content does not differ between SAF inflammatory activity grades. Level of significance: *p* = 0.95 (Kruskall-Wallis test). (C) Pancreatic fat content is unrelated to hepatic fibrosis stage. Level of significance: *p* = 0.23 (Mann-Whitney *U* test). Dashed line represents the FPD cut-off of 6.2%. FPD, fatty pancreas disease.

Pancreatic PDFF did not differ between participants with increasing liver steatosis grades (Fig. 3A), nor was it different across liver SAF inflammatory activity grades as a measure of steatohepatitis (Fig. 3B). Moreover, pancreatic PDFF did not differ between participants with or without advanced hepatic fibrosis (F ≥3) (Fig. 3C).

#### Pancreatic T1

Pancreatic T1 relaxation time did not differ between participants with increasing liver steatosis grades (ANOVA, *p* = 0.73), increasing SAF inflammatory activity grades (ANOVA, *p* = 1.00), or between participants with/without advanced liver fibrosis (F ≥3) (t-test, *p* = 0.33).

#### Pancreatic IVIM-DWI

**Fig. 4:**
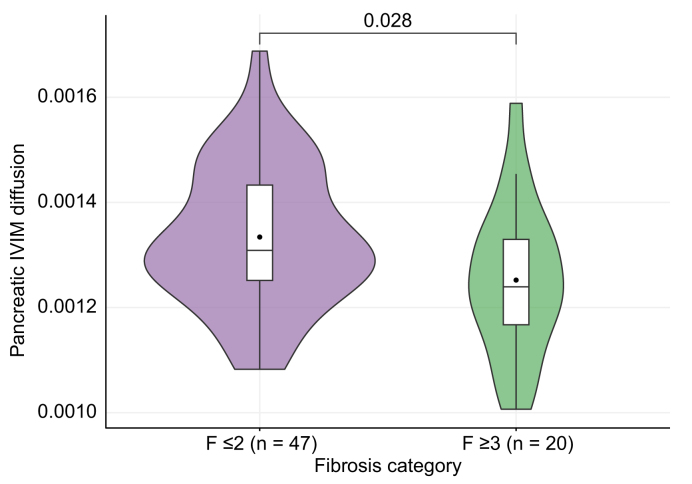
Pancreatic IVIM diffusion is lower in individuals with advanced hepatic fibrosis. Level of significance: *p* = 0.028 (t-test). IVIM, intravoxel incoherent motion.

IVIM-D was not related to liver steatosis grades (ANOVA, *p* = 0.10) or SAF inflammatory activity grades (ANOVA, *p* = 0.17). However, when comparing participants on fibrosis severity, we identified significantly lower IVIM-D values in those with advanced fibrosis (Fig. 4).

IVIM-*f* was not related to liver steatosis grades (Kruskal-Wallis, *p* = 0.56), SAF inflammatory activity grades (Kruskal-Wallis, *p* = 0.11), or hepatic fibrosis stages (Kruskal-Wallis, *p* = 0.73).

### Pancreatic MRI parameters are unrelated to exocrine function

When evaluating whether pancreatic MRI outcomes were related to exocrine function, our findings showed that pancreatic PDFF did not differ between individuals with exocrine insufficiency and those with normal fecal elastase levels (Mann-Whitney *U* test, *p* = 0.95). Similarly, neither pancreatic T1 (t-test, *p* = 0.74), IVIM-D (t-test, *p* = 0.39) or IVIM-*f* (Mann-Whitney *U* test, *p* = 0.31) were different between individuals with exocrine insufficiency and those with normal fecal elastase levels.

## Discussion

This study finds no support for a relation between IPFD and hepatic steatosis in individuals with MASLD. Instead, we uncovered other significant findings. Firstly, we observed an association between pancreatic T1 relaxation time on MRI, a marker of inflammation and fibrosis,^25^ and glycemic dysregulation. Our cohort consisted of individuals with MASLD, of whom 40% had type 2 diabetes mellitus and 88% exhibited insulin resistance (HOMA-IR >2.0). This cohort represents a group of relatively young, hyperinsulinemic patients with MASLD developing hepatic fibrosis who have not (yet) reached a state of severe pancreatic beta-cell failure and insulinopenia. Additionally, we identified a relationship between pancreatic IVIM-D (as a proxy of pancreatic collagen fraction) and hepatic fibrosis. The results from this study support the notion that pancreatic fibro-inflammation in individuals with MASLD may be more related to glucotoxicity than to lipotoxicity, a key process in MASLD.

The prevalence of FPD in this cohort was 37.3%. Wang *et al.* reported a larger proportion of 67% in a population with MASLD^35^ and Uygun reported 51%.^36^ However, both these studies used ultrasound for the diagnosis of FPD, which is likely less accurate than MRI. Also, they did not use a cut-off for normal IPFD, which may have led to overdiagnosis. In a large community cohort of 685 healthy volunteers, Wong *et al.* found an FPD prevalence of 16.1% (95% CI 13.3–18.8%) in the general population using MRI.^37^

IPFD was not related to hepatic steatosis as assessed by MRI and liver histology. This finding aligns with some previous works.^38^^,^^39^ However, other previously conducted studies have suggested that fat accumulation in the pancreas does have negative consequences on liver disease severity in MASLD.^15^^,^^18^^,^^19^^,^^40^ There are several potential explanations to account for the differences with our findings. For instance, some of these studies used ultrasound to diagnose FPD,^19^ which is unable to distinguish between IPFD and peripancreatic fat tissue. The studies that did use MRI-PDFF consisted of smaller cohorts than our study.^18^^,^^40^ Moreover, the association between FPD and MASLD in these studies may have been mediated by obesity, as the association disappeared in several studies when correcting for BMI^15^ or visceral fat volume.^40^ In our cohort, consisting of individuals with overweight or obesity only, we did not see this mediation. Alternatively, it is possible that the timing of intrapancreatic and intrahepatic fat deposition differs, which could also explain the lack of correlation between the two in this study. Still, our results align with a study that evaluated the reduction in fat content of the liver and pancreas after bariatric surgery, which showed that the reduction of fat in both of these tissues was unrelated.^41^ Together, this suggests that hepatic and pancreatic fat are likely separately occurring entities rather than related processes.

In contrast to several previously conducted studies, we did not find a negative association between pancreatic fat content and glucose regulation.^4^^,^^12^^,^^42^ Again, this may be explained by the differences in methods, sample sizes and cohort characteristics. Notably, we found that pancreatic T1 relaxation time correlated with markers of glycemic control: pancreatic T1 relaxation time was significantly higher in individuals with type 2 diabetes and correlated positively with fasting glucose and HbA1c values. In the liver, T1 mapping is used to assess the extent of hepatic fibrosis and inflammation. Higher T1 relaxation time is indicative of increased extracellular fluid content, a characteristic of fibrosis and inflammation.^43^ Furthermore, studies have shown that iron-corrected T1 (cT1) can distinguish between isolated steatosis and MASH.^44^ In chronic pancreatitis, pancreatic T1 increases progressively with worsening stages.^24^ Our results thus indicate the presence of inflammatory processes in the pancreas of individuals with impaired glucose homeostasis. Although we did not observe a relationship between pancreatic T1 and PIR, the trend towards an inverse correlation with C-peptide suggests reduced beta-cell function in individuals with MASLD and pancreatic fibro-inflammation. Since we found no such correlation between pancreatic T1 and fasting insulin levels, we investigated whether suppressed fasting insulin clearance was already occurring in this group. However, pancreatic T1 and the C-peptide to insulin molar ratio (as a proxy for insulin clearance) were not correlated. Of note, pancreatic T1 has previously been proposed as a potential diagnostic marker for impaired glucose tolerance based on its strong correlation with HbA1c.^45^ Our findings align with previous reports showing no association between pancreatic fat and beta-cell function.^42^^,^^46^ They suggested that other factors besides pancreatic fat may contribute to further decline once diabetes has manifested. The results from our study provide evidence that pancreatic fibro-inflammation may indeed be one of those influencing factors.

With regard to IVIM-DWI imaging, the median pancreatic IVIM-*f* of 13.3% in our cohort was lower than has been reported in healthy controls (around 24%).^25^^,^^47^ It has been shown that IVIM-*f* decreases significantly in auto-immune pancreatitis to around 10%.^47^^,^^48^ Thus, the low IVIM-*f* in this cohort also suggests inflammatory processes in the pancreas of individuals with MASLD. As noted in the introduction, IVIM-*f* has been correlated with vessel density. Infiltration of inflammatory cells and an increase in pancreatic fibrosis, as seen in auto-immune pancreatitis, could lead to reduced vessel density and thus explain reduced perfusion fractions.^48^ Regarding pancreatic IVIM-D, the observed lower values in individuals with advanced liver fibrosis indicate a reduction in pancreatic diffusion, suggesting the presence of pancreatic fibrosis in individuals with advanced fibrotic stages of MASLD.^25^ Together, these IVIM-DWI findings suggest that indeed other pancreatic processes besides IPFD may occur that interrelate to liver disease in individuals with MASLD.

This work has several strengths. First, the use of a multiparametric MRI scanning protocol in addition to tandem-read liver biopsies evaluated by two pathologists, meant that our cohort of individuals with MASLD were well characterized. Also, the participants included represent a wide range of the MASLD severity spectrum, with mild to severe steatosis and fibrosis scores. For the assessments of IPFD, ROIs were placed in three parts of the pancreas to ensure representativeness.

This study also has some limitations. For pancreatic T1 analyses, results should be interpreted as exploratory due to the limited size of the available subgroup. Also, it should be noted that the MRI methods, especially the T1 and IVIM-DWI sequences, are relatively new. No histologic confirmation in pancreatic tissue is available, as pancreatic biopsies are not performed. However, these imaging methods provide new opportunities to evaluate inter-organ disease processes, as this work demonstrates. Further studies should focus on the pathophysiological bases of our observations: what causes the increased T1 and altered IVIM-DWI properties of the pancreas? Which pancreatic cell type underlies these changes? Do these alterations occur before aggravation of MASLD severity or as a result of it?

In conclusion, our cross-sectional study supports the notion that in obesity and MASLD, the occurrence of lipid accumulation in the liver and pancreas are not closely related, potentially indicating two distinct processes during disease development in this patient group. The correlations between prolonged pancreatic T1 relaxation time, as a measure for pancreatic inflammation and fibrosis, and parameters of glycemic dysregulation suggest that glucotoxicity may be a relevant process in the pancreas of individuals with MASLD, potentially more so than lipotoxicity.

## Financial support

MN is supported by a personal 10.13039/501100001826ZONMW-VICI grant 2020 (09150182010020). A.G.H. is supported by the Amsterdam UMC Fellowship grant, an Amsterdam UMC Innovation grant, two grants from the Dutch Gastroenterology Foundation MLDS and two grants from Holland∼Health TKI-PPP. These funding sources had no involvement in the preparation of this work.

## Authors’ contributions

A.L.M., A.G.H. and D.C. devised the project. A.G.H., M.N. and U.B. designed the cohort study. A.L.M., A-M.vD., V.H., K.v.S., S.D. and D.Z. performed the patient visits. S.v.d.B. and E.S. performed the MRI scanning. A.L.M., N.W. and M.T. performed the analysis. E.S., J.H.R. and O.G-C. designed analysis pipelines and aided in interpreting the results. M.D. and J.V. scored the liver biopsies. A.L.M., N.W. and A.G.H. afted the manuscript. All authors provided feedback on the writing and accepted the final version of the manuscript.

## Data availability statement

The data that support the findings of this study are available from the corresponding author, A.L.M., upon reasonable request.

## Conflict of interest

ES is employed by Perspectum Ltd., who provided some of the MRI protocols used for the multiparametric MRI data reported in this paper. All other authors declare no conflicts of interest.

Please refer to the accompanying ICMJE disclosure forms for further details.
