# Supplementary material for: Intrapancreatic fat deposition is unrelated to liver steatosis in metabolic dysfunction-associated steatotic liver disease
Source: JHEP Rep. 2024 Jan 1;6(3):100998. doi: 10.1016/j.jhepr.2023.100998 (PMC10877191; doi:10.1016/j.jhepr.2023.100998)
Supplement: Multimedia component 1 [file mmc1.pdf]

Intrapancreatic fat deposition is unrelated to liver steatosis in metabolic dysfunction-associated steatotic liver disease

Anne Linde Mak, Nienke Wassenaar, Anne-Marieke van Dijk, Marian Troelstra, Veera Houttu, Koen van Son, Stan Driessen, Diona Zwirs, Sandra van den Berg-Faay, Elizabeth Shumbayawonda, Jurgen Runge, Michail Doukas, Joanne Verheij, Ulrich Beuers, Max Nieuwdorp, Djuna L. Cahen, Aart Nederveen, Oliver Gurney-Champion, Adriaan Holleboom

Table of contents

Fig. S1……………………………………………………………………….........................2

Fig. S2……………………………………………………………………….........................4

Fig. S3……………………………………………………………………….........................5

Table S1……………………………………………………………………….......................7

Table S2……………………………………………………………………….......................9

Supplementary references……………………………………………………………..9

Fig. S1

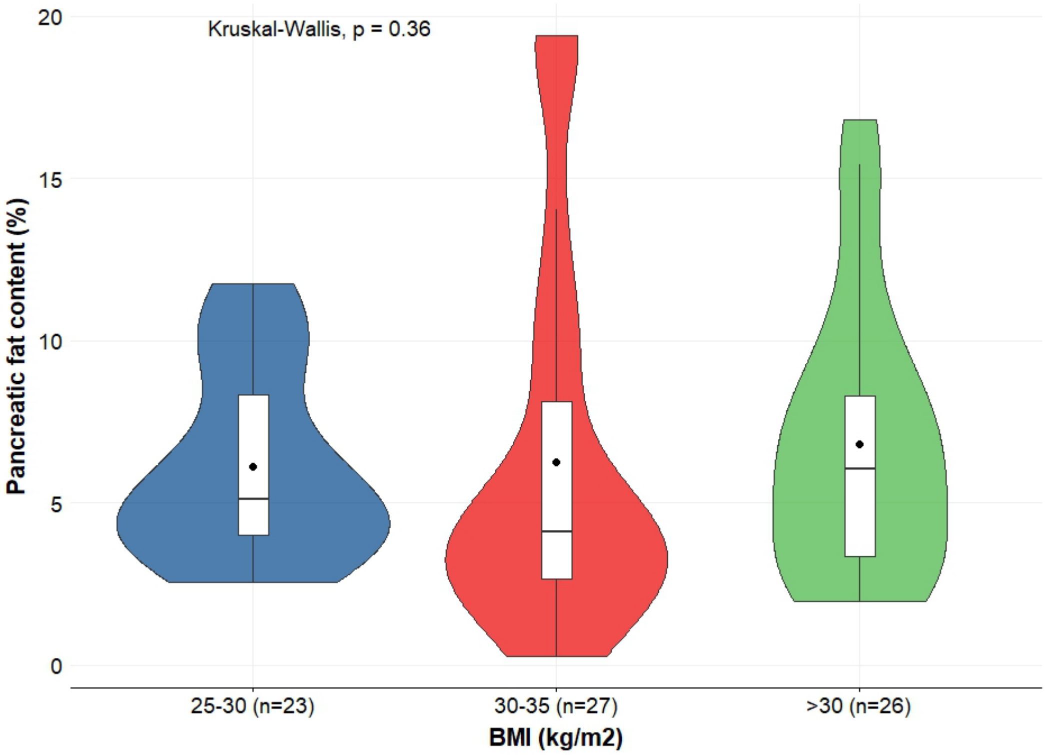

Kruskal-Wallis, p = 0.36
Pancreatic fat content (%)
20
15
10
5
0
25-30 (n=23)
30-35 (n=27)
>30 (n=26)
BMI (kg/m2)

1A:

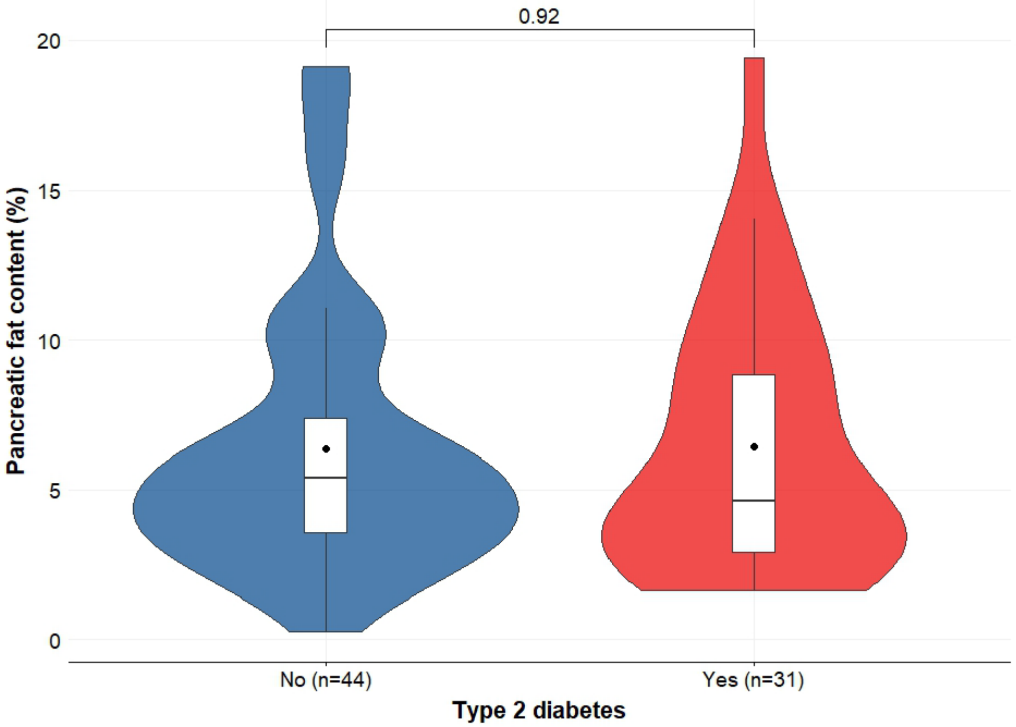

0.92
Pancreatic fat content (%)
20
15
10
5
0
No (n=44)
Yes (n=31)
Type 2 diabetes

1B:

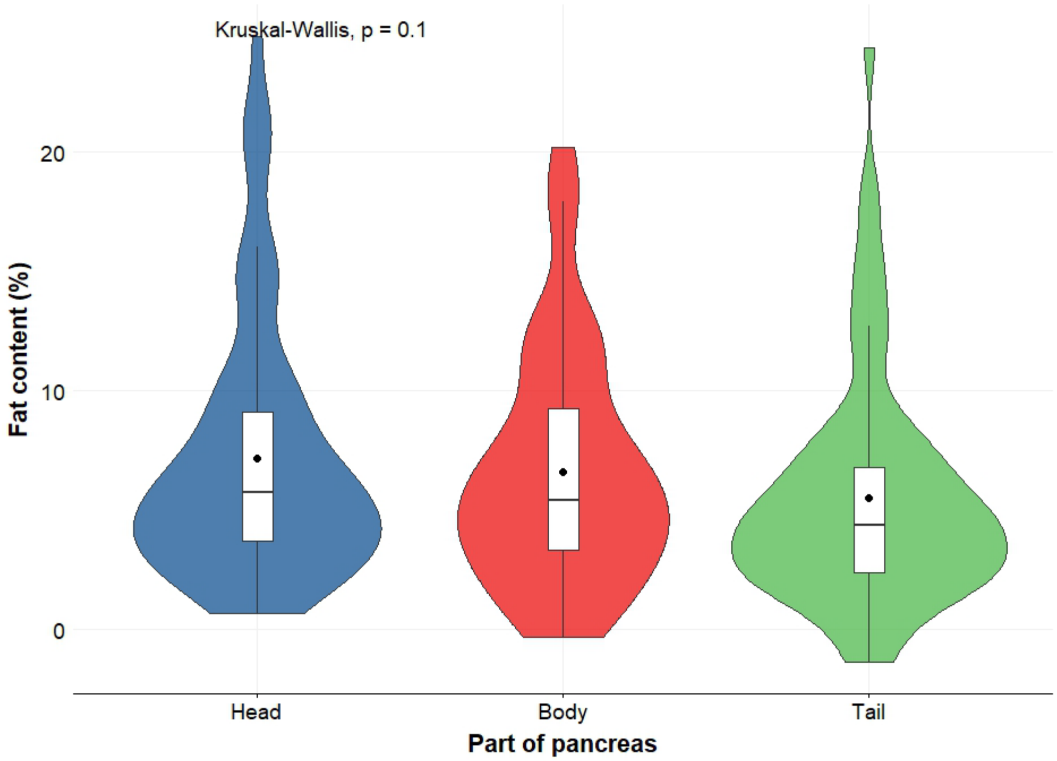

1C:

Fig. S1. **Pancreatic fat content is not influenced by BMI, type 2 diabetes status or pancreatic region measured.** (A) BMI categories. Level of significance: $p = 0.36$ (Kruskal-Wallis test). (B) Type 2 diabetes mellitus. Level of significance: $p = 0.92$ (Mann-Whitney *U* test). (C) Pancreatic regions. Level of significance: $p = 0.1$ (Kruskal-Wallis test).

Fig. S2

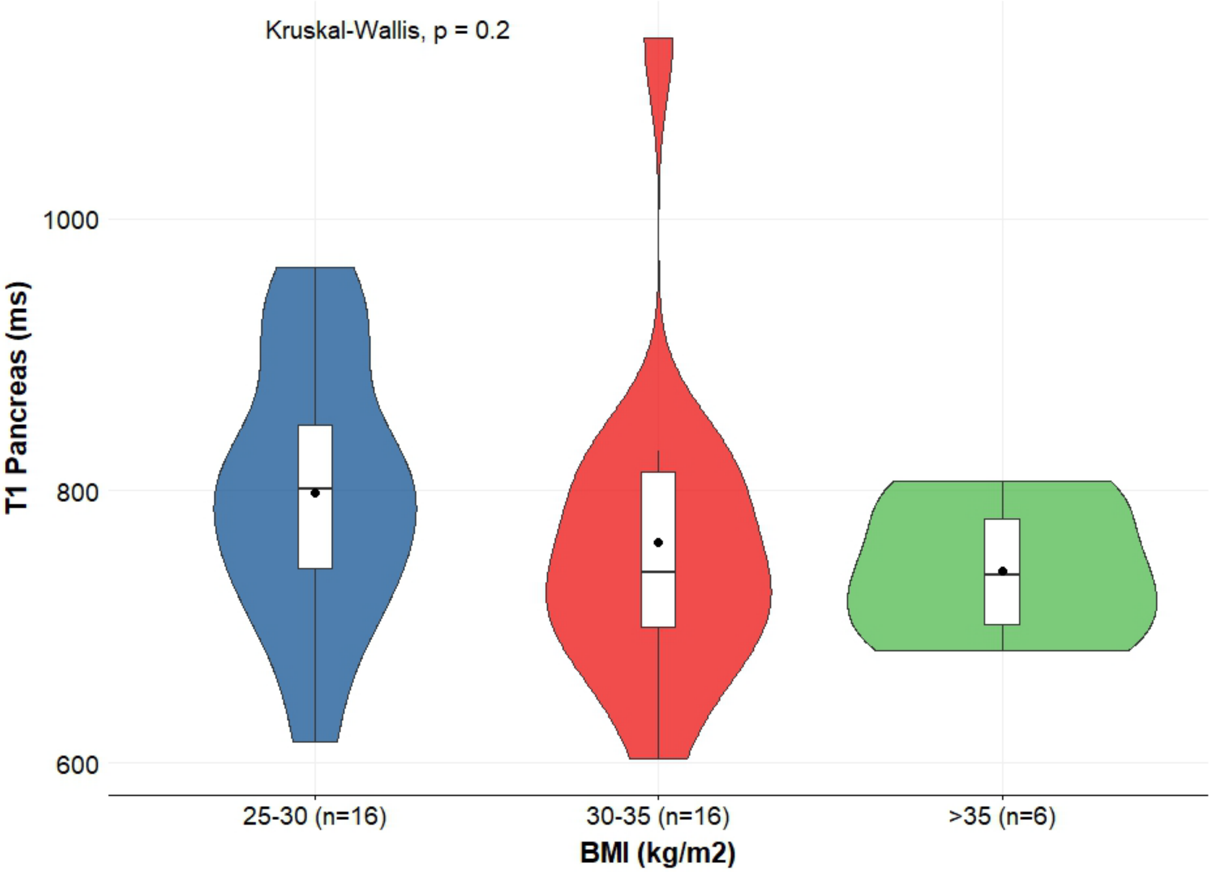

Fig. S2. Pancreatic T1 relaxation time does not differ between participants with in different BMI categories. Level of significance: $p = 0.11$ (ANOVA).

Fig. S3

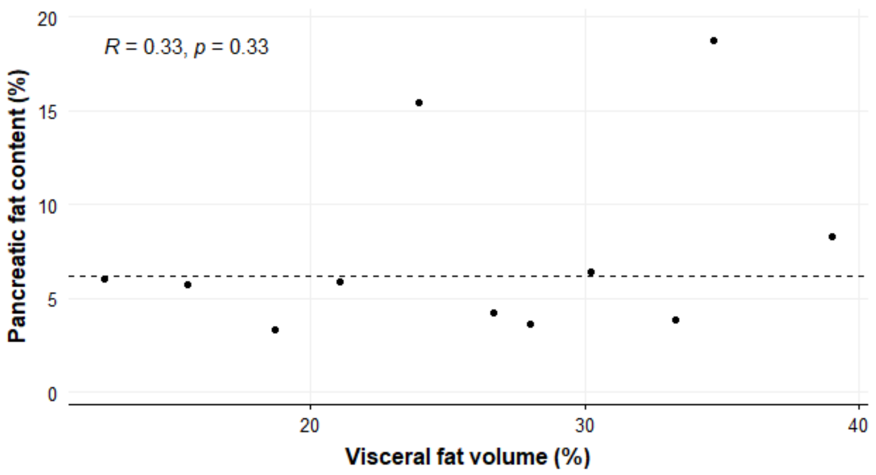

3A:

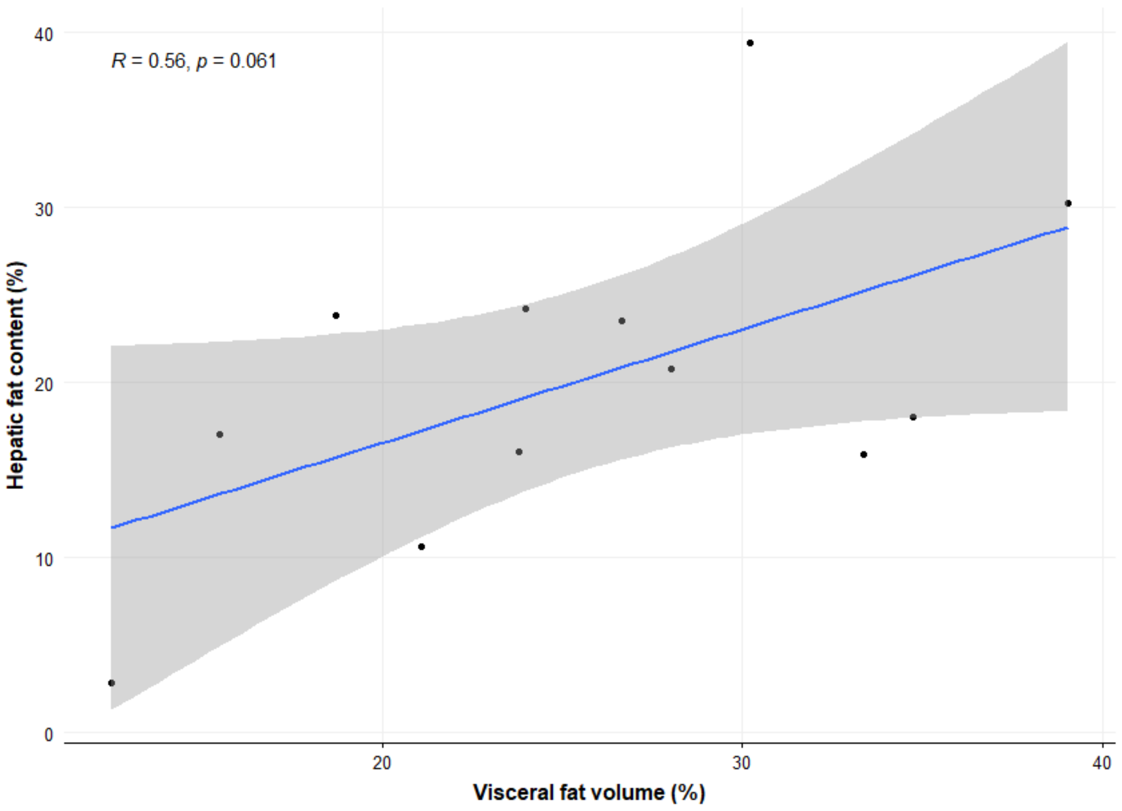

3B:

Fig. S3. Correlations between pancreatic and hepatic PDFF and visceral fat percentage in a subgroup of 11 participants. (A) Pancreatic fat content was not correlated with visceral fat

percentage. Level of significance: $p = 0.33$ (Pearson). Dotted line represents FPD cut-off of 6.2%. (B) Hepatic fat content tended to be correlated with visceral fat percentage. Level of significance: $p = 0.061$ (Pearson).

Table S1. Cohort characteristics by sex. Hepatic steatosis, lobular inflammation and fibrosis are graded according to the SAF scoring system (1). Data are presented as mean (SD), median [IQR], or count (percentage).

		**Female**	**Male**	**p**
	n	31	45	
	Age	51.00 (11.61)	44.98 (14.50)	0.058
	BMI (kg/m^2)	32.64 [29.34, 34.72]	33.08 [29.70, 36.90]	0.251
	Type 2 diabetes (%)	12 (38.7)	19 (42.2)	0.945
	HbA1c (mmol/mol)	40.00 [38.00, 53.00]	43.00 [36.00, 53.00]	0.850
	Fasting glucose (mmol/L)	6.10 [5.50, 7.50]	6.20 [5.40, 8.00]	0.916
	Fasting insulin (pmol/L)	120.00 [96.00, 148.00]	127.00 [77.00, 202.00]	0.409
	AST (U/L)	38.00 [33.50, 63.00]	42.00 [37.00, 53.00]	0.665
	ALT (U/L)	59.00 [41.00, 81.50]	62.00 [52.00, 100.00]	0.238
	gGT (U/L)	59.00 [30.00, 89.50]	66.00 [49.00, 91.00]	0.241
	ALP (U/L)	101.68 (35.95)	74.76 (21.86)	**<0.001**
**MRI outcomes**				
	PDFF Pancreas	4.21 [3.31, 8.50]	5.17 [3.52, 8.30]	0.611
	T1 Pancreas	801.00 [732.00, 829.00]	742.00 [691.00, 791.00]	0.060
	IVIM-D	0.00 (0.00)	0.00 (0.00)	0.643

	IVIM-*f*	13.20 [11.28, 15.07]	13.30 [11.99, 15.97]	0.456
	PDFF Liver	19.82 (8.12)	16.29 (8.53)	0.075
	cT1 Liver	928.25 (104.96)	878.18 (98.68)	0.050
**Histology scoring of MASLD**				
**Steatosis**	0	0 (0.0)	3 (6.7)	0.423
	1	9 (29.0)	14 (31.1)	
	2	12 (38.7)	18 (40.0)	
	3	10 (32.3)	10 (22.2)	
**Inflammatory activity**	0	2 (6.5)	2 (4.4)	0.649
	1	8 (25.8)	13 (28.9)	
	2	13 (41.9)	23 (51.1)	
	3	7 (22.6)	7 (15.6)	
	4	1 (3.2)	0 (0.0)	
**Fibrosis**	0	0 (0.0)	3 (6.7)	0.103
	1	7 (22.6)	2 (4.4)	
	2	14 (45.2)	25 (55.6)	
	3	8 (25.8)	11 (24.4)	
	4	2 (6.5)	4 (8.9)	

Table S2. Number of cardiometabolic criteria of included participants. Criteria are defined according to the multi-society Delphi consensus statement on new fatty liver disease nomenclature (2).

Number of cardiometabolic criteria	Number of participants
**1**	3
**2**	8
**3**	18
**4**	18
**5**	29

Supplementary references

1. Bedossa P, Poitou C, Veyrie N, Bouillot JL, Basdevant A, Paradis V, et al. Histopathological algorithm and scoring system for evaluation of liver lesions in morbidly obese patients. Hepatology. 2012 Nov;56(5):1751–9.
2. Rinella ME, Lazarus JV, Ratziu V, et al. A multisociety Delphi consensus statement on new fatty liver disease nomenclature. J Hepatol. 2023;S0168-8278(23)00418-X. doi:10.1016/j.jhep.2023.06.003
