# Supplementary material for: Intrapancreatic fat deposition is unrelated to liver steatosis in metabolic dysfunction-associated steatotic liver disease
Source: JHEP Rep. 2024 Jan 1;6(3):100998. doi: 10.1016/j.jhepr.2023.100998 (PMC10877191; doi:10.1016/j.jhepr.2023.100998)
Supplement: Multimedia component 2 [file mmc2.docx]

**JHEP Reports**

**CTAT methods**

Tables for a “Complete, Transparent, Accurate and Timely account” (CTAT) are now mandatory for all revised submissions. The aim is to enhance the reproducibility of methods.

- Only include the parts relevant to your study
- Refer to the CTAT in the main text as ‘Supplementary CTAT Table’
- Do not add subheadings
- Add as many rows as needed to include all information
- Only include one item per row

**If the CTAT form is not relevant to your study, please outline the reasons why:**

|  |
| --- |

- 1. **Antibodies**

| **Name** | **Citation** | **Supplier** | **Cat no.** | **Clone no.** |
| --- | --- | --- | --- | --- |
|  |  |  |  |  |

- 1. **Cell lines**

| **Name** | **Citation** | **Supplier** | **Cat no.** | **Passage no.** | **Authentication test method** |
| --- | --- | --- | --- | --- | --- |
|  |  |  |  |  |  |

- 1. **Organisms**

| **Name** | **Citation** | **Supplier** | **Strain** | **Sex** | **Age** | **Overall n number** |
| --- | --- | --- | --- | --- | --- | --- |
| Humans | n/a | n/a | n/a | 31 women  45 men | Average 47.4 years (SD 13.6) | 76 |

- 1. **Sequence based reagents**

| **Name** | **Sequence** | **Supplier** |
| --- | --- | --- |
|  |  |  |

- 1. **Biological samples**

| **Description** | **Source** | **Identifier** |
| --- | --- | --- |
| Amsterdam UMC MASLD Cohort (ANCHOR) Study | Liver biopsies, blood and stool samples at baseline;  All collected between 2018-2023 | Trial registry number NTR7191.  Samples stored at AMC Biobank: ANCHOR |

- 1. **Deposited data**

| **Name of repository** | **Identifier** | **Link** |
| --- | --- | --- |
|  |  |  |

- 1. **Software**

| **Software name** | **Manufacturer** | **Version** |
| --- | --- | --- |
| R | R core team | 4.2.1 |
| LiverMultiScan | Perspectum | n/a |
| Python | Python Software Foundation | 3.6.4 |
| Pytorch | Linux Foundation | 0.4.1 |

- 1. **Other (*e.g*. drugs, proteins, vectors etc.)**

|  |  |  |
| --- | --- | --- |
|  |  |  |

- 1. **Please provide the details of the corresponding methods author for the manuscript:**

| A.G. Holleboom  Department of Vascular Medicine  Amsterdam University Medical Centers, Amsterdam, The Netherlands.  a.g.holleboom@amsterdamumc.nl |
| --- |

**2.0 Please confirm for randomised controlled trials all versions of the clinical protocol are included in the submission. These will be published online as supplementary information.**

|  |
| --- |
